# Cytogenetically normal uterine leiomyomas without *MED12*-mutations – a source to identify unknown mechanisms of the development of uterine smooth muscle tumors

**DOI:** 10.1186/s13039-014-0088-1

**Published:** 2014-11-29

**Authors:** Carsten Holzmann, Dominique Nadine Markowski, Dirk Koczan, Wolfgang Küpker, Burkhard Maria Helmke, Jörn Bullerdiek

**Affiliations:** Institute of Medical Genetics, University Rostock Medical Center, Ernst-Heydemann-Strasse 8, D-18057 Rostock, Germany; Center of Human Genetics, University of Bremen, Leobener Strasse ZHG, D-28359 Bremen, Germany; Institute for Immunology, University Medicine, University of Rostock, Schillingallee 70, D-18057 Rostock, Germany; Center for Minimal Invasive Gynecology, Endometriosis, and Reproductive Medicine, Klinikum Mittelbaden, Balger Str. 50, 76532 Baden-Baden, Germany; Institute of Pathology, University of Heidelberg, Heidelberg, Germany; Present address: Institute of Pathology, Elbe Kliniken, Klinikum Stade, Bremervörder Str. 111, D- 21682 Stade, Germany

**Keywords:** Uterine leiomyomas, Smooth muscle, Driver mutations, Chromothripsis

## Abstract

**Background:**

Recent findings on genetic changes in uterine leiomyomas suggest these benign tumors being a heterogeneous group of diseases in terms of molecular pathogenesis with those showing karyotype alterations as well as those characterized only by cytogenetically invisible mutations of *mediator subcomplex 12 (MED12).* Herein, five uterine leiomyomas (UL) with an apparently normal karyotype that lacked *MED12*-mutations were investigated by copy number variation arrays along with their matching myometrium to search for small genomic imbalances.

**Results:**

Of five tumors one showed chromothripsis-like phenomena with numerous gains and losses of small segments mainly clustered to five chromosomal regions i.e. 2p14-2pter, 2q33.1-2q37.3, 5q31.3-5qter,11q14.1-11qter, and 18p11.21-18q2.3. Apparently, these cells had escaped detection by classical cytogenetics. Histologically, the tumor presented as a cellular leiomyoma with extended hyalinization. Of the remaining four tumors, one had a small intragenic deletion of the *HMGA2* gene that was lacking in the corresponding myometrium. The other three tumors did not show relevant copy number alterations at all.

**Conclusions:**

Overall, the results suggest that leiomyomas with an apparently normal karyotype based on classical cytogenetics and lacking *MED12* mutations represent a heterogeneous group of diseases. While the *HMGA2* deletion detected in one of the tumors likely represents the driver mutation and, due to its size, has escaped detection by classical cytogenetics, the extended genomic imbalances detected in one of the other cases cannot be overlooked by this method suggesting an inability of the affected cells to divide in vitro. Of particular interest in that case is the occurrence of so-called “chromothripsis” or “firestorms” without involvement of the loci of common chromosomal rearrangements in UL, as e.g. 12q14 ~ 15 and 6p21. While chromothripsis was initially described as a hallmark of malignancy, the etiology and significance of this phenomenon in benign tumors still remain obscure. In uterine smooth muscle tumors, these changes per se do not indicate malignancy.

## Background

Uterine leiomyomas are highly frequent gynaecological tumors. Genetic changes confined to the tumor tissue and lacking in adjacent myometrial tissue offer a highly valuable tool to shed light on the molecular pathogenesis of the disease. Moreover, they may help to understand pathways of malignant transformation either occurring in pre-existing leiomyomas or following an independent route of tumorigenesis. Recent data suggest that both pathways may account for a considerable percentage of leiomyosarcomas. The ongoing discussion on the risk of iatrogenic metastatic spreading of incidental leiomyosarcomas by powermorcellation underscores the unmet medical need to define and find suitable biomarkers in that field.

The vast majority of uterine leiomyomas (UL) either display clonal karyotype aberrations detected by classical cytogenetics or mutations of *Mediator Subcomplex 12* (*MED12*) [1,2], or both. Besides rare cases of germline mutations of the *Fumarate Hydratase* (*FH*) gene [3] associated with an early onset of cutaneous and uterine UL as well as with renal cancer or of *Col* genes [4], two frequent types of independent somatic driver mutations have been deduced i.e. rearrangements of high mobility group protein gene 2 (*HMGA2*) [5] and, much more frequently those affecting *MED12* [1]. Apparently, these both mutations do not overlap with each other [2,4] and seem to be associated with different tumor sizes [1,2] as well as with their presence as multiple versus solitary tumors (Markowski et al., in press). Moreover, *MED12* mutated UL but not those with *HMGA2* rearrangements apparently display strong growth disadvantages *in vitro* [6] while, in rare cases, they may undergo malignant transformation following an UL-STUMP (smooth muscle tumor of uncertain malignant potential)-leiomyosarcoma sequence as witnessed by leiomyosarcomas and STUMP carrying *MED12* mutations [7-11]. However, having identified those leiomyomas carrying *MED12* mutations or/and cytogenetic abnormalities, there remains a small percentage of 10-15% of leiomyomas without these abnormalities. Recently, we have described one such tumor displaying nearly genome-wide uniparental disomy with some additional genetic alterations that was histologically classified as an epithelioid leiomyomas [12]. This case encouraged us to investigate further leiomyomas remaining without *MED12* mutations but with an apparently normal karyotype.

Herein, we have examined five of these cases along with corresponding myometrial tissue by CNV (copy number variation) arrays. The results revealed genetic alterations in 2/5 tumors which in one of the cases only corresponds to with the locus of a previously identified driver mutation. In another case, the analysis leads to the identification of gross genetic rearrangements that apparently had escaped detection by conventional cytogenetics. The significance of these findings in term of molecular pathogenesis is discussed.

## Results

Five uterine leiomyomas (UL) were investigated by CNV-arrays along with their matching myometrium (Table 1). All these UL lacked karyotypic abnormalities as detected by classical cytogenetics as well as *MED12* mutations in the hot spot region as initially described by Mäkinen et al..

**Table 1:** **Patients age, size of the tumor, and karyotype after classical cytogenetics of five randomly selected uterine leiomyomas lacking cytogenetically detectable karyotype alterations as well as** ***MED12*** **-mutation of the “leiomyomas-type” as first described by Mäkinen et al.** [1]

**Case**	**Patients age**	**Tumorsize [cm]**	**Karyotype**
My 605	45	n.d.	46,XX [22]
My 620-2	45	3.5	46,XX [16]
My 678	43	1.3	46,XX [20]
My 708-2	42	5	46,XX [20]
My706	43	3.5	46,XX [18]

**Figure 1:**
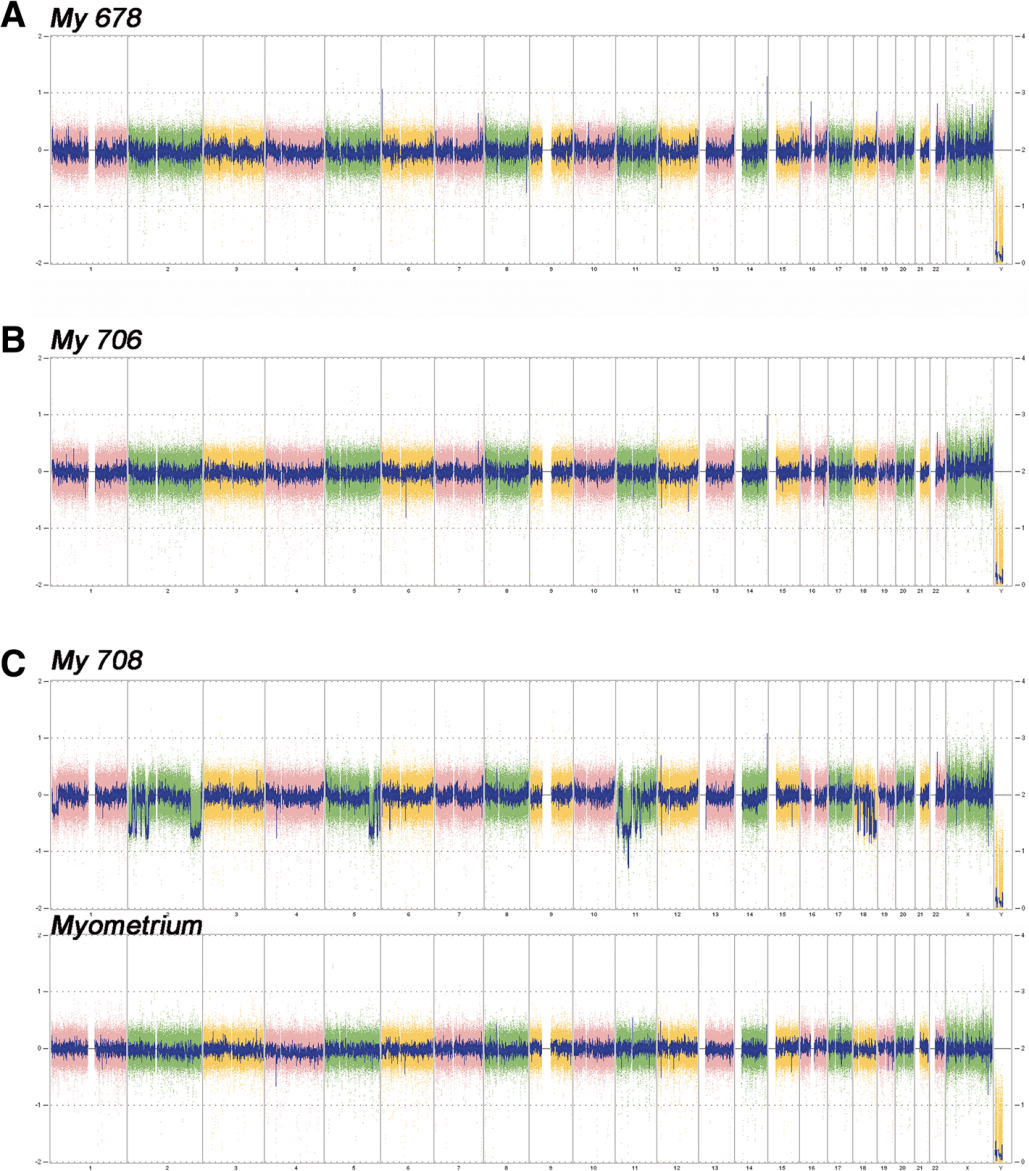
**Copy number variation (CNV)-array analysis of two leiomyomas without gross copy number alterations (A: My 678, B: My 706) and one leiomyomas showing multiple genomic losses within five genomic regions (C: My 708) that were absent in the matching myometrial tissue (C: myometrium).**

**Figure 2:**
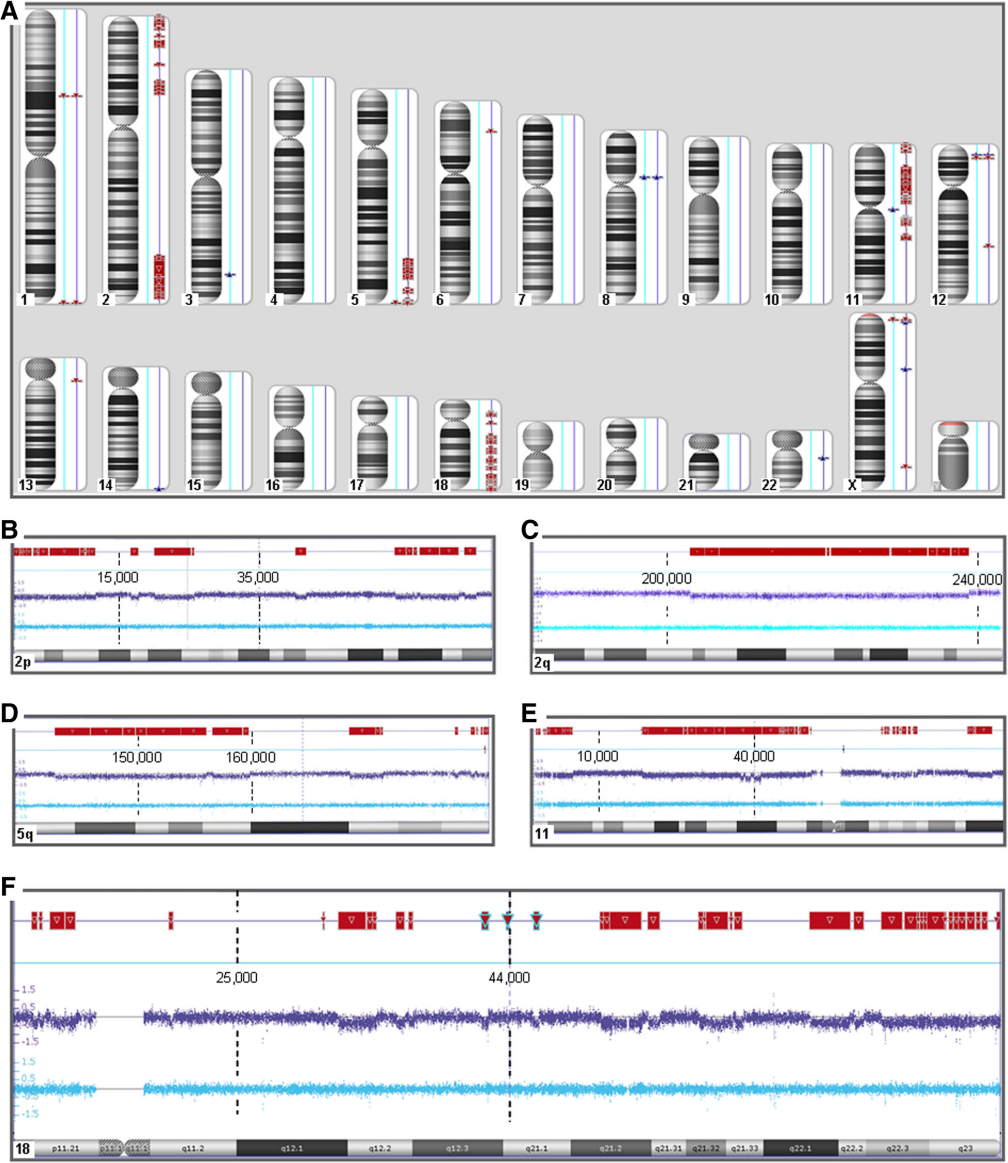
**Leiomyoma (#708) with extended copy number alterations confined to four distinct chromosomal regions and the whole chromosome 18, respectively. A**: Genomic view of the case showing for each chromosome the ideogram, the myometrial tissue (light blue line), and the leiomyomas (dark blue line). Red blocks represent deletions. **B-F**: Detailed view of the five parts of the genome showing numerous losses, i.e. 2p14 → 2pter, 2q33.1→2qter, 5q31.3→5qter, 11q14.1→11qter, and 18p11.21→18q22.3. Within each diagram the two lines above the ideogram represent myometrial tissue (light blue) and the leiomyoma (dark blue), respectively. Dashed lines and numbers represent the map positions according to Hg19 (NCBI Build 37 reference sequence). In the top line, the positions of deletions are shown as red blocks.

**Figure 3:**
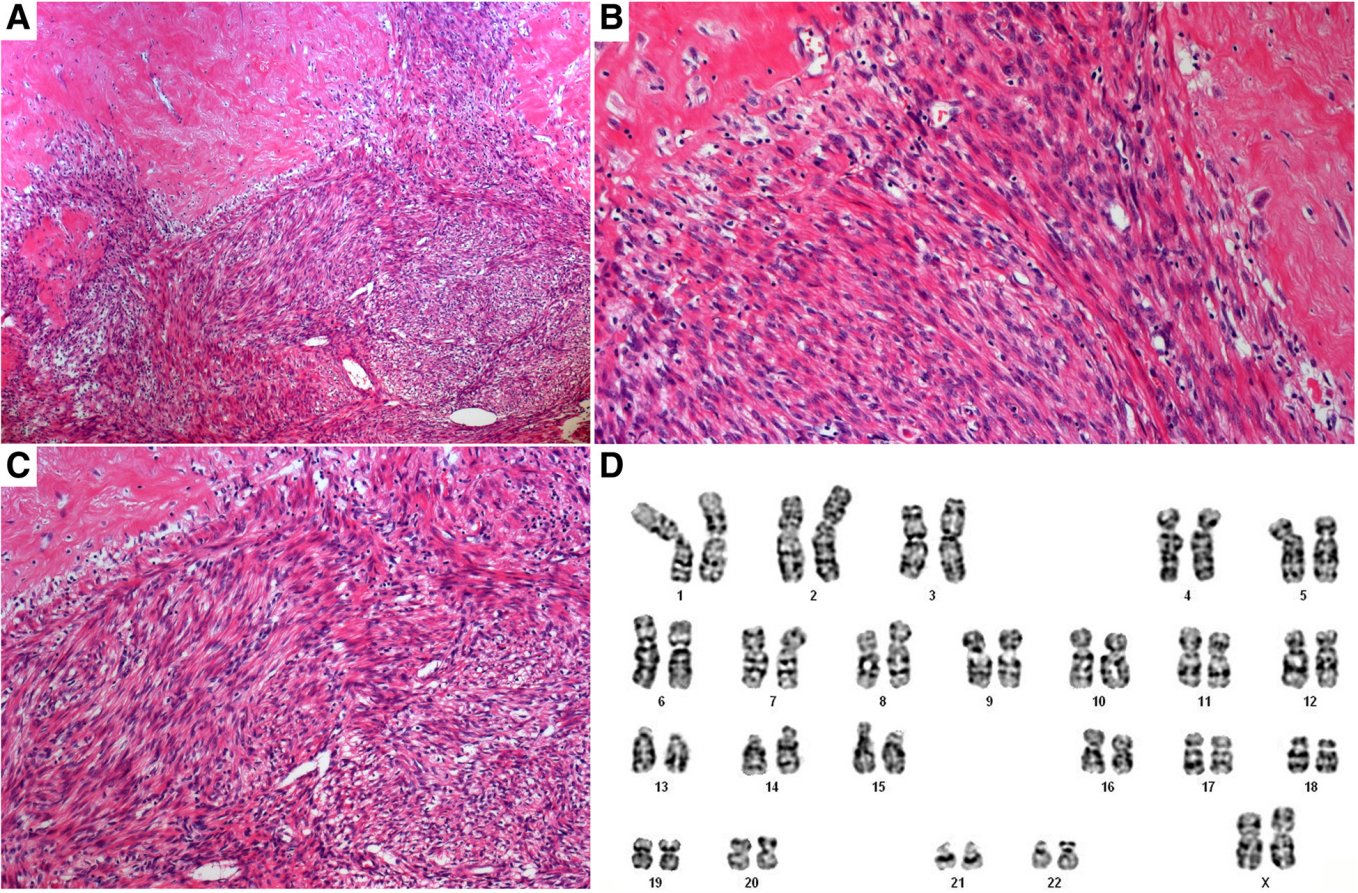
**Histologic appearance and cytogenetic analysis of tumor 708–2.** Histologic appearance after H&E staining reveals a cell-rich leiomyoma with extended hyalinization. **A**: Overview. **B,C**: Histology at larger magnification. **D**: Representative G-banded karyotype at a level of approximately 350 bands/haploid set.

Accordingly, four of the tumors did not reveal gross copy number alterations after CNV-analysis (Figure 1A, B). In contrast, in one tumor (My 708-2) numerous deletions were seen while the myometrial tissue did not show these deletions (Figures 1C and 2A). Interestingly, these copy number alterations were mainly restricted to a total of five chromosomal regions i.e. 2p14 → 2pter, 2q33.1 → 2q37.3, 5q31.3 → 5qter, 11q14.1 → 11qter, and 18p11.21 → 18q2.3 (Figure 2B-F). Across these five parts of the genome it was possible to detect at least 145 distinct regions of copy number alterations ranging in size between 43 and 13,647,027 kbp. On the other hand, in that case classical cytogenetics on a 275 to 550 bands/haploid set-level revealed an apparently normal karyotype 46,XX in all 20 metaphases examined (Figure 3A). Histologically, the tumor presented as a cellular leiomyoma with large areas of hyalinization (Figure 3B, C).

**Figure 4:**
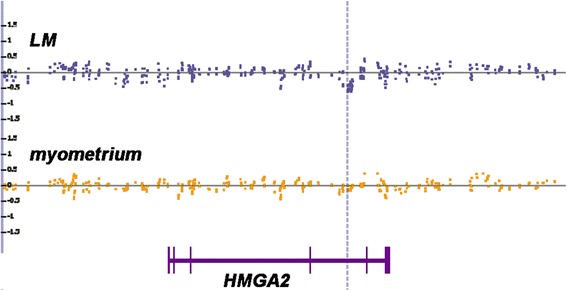
**Copy number alterations indicating a small intragenic deletion within the** ***HMGA2*** **gene, case #706, dotted line.** LM and blue squares: leiomyomas tissue, yellow squares: myometrial tissue. Bottom line: genomic structures of the *HMGA2* gene with vertical bars indicating exons based on [5].

Aimed at the detection of small copy number alterations we have then performed a detailed comparison of the copy number profiles of leiomyomas vs. myometrial tissue in the remaining four cases. Interestingly, one of the tumors (My 706) showed a small deleted region within intron 4 of the *HMGA2* gene. This deletion had a size of approximately 6 kbp and was lacking in the corresponding normal smooth muscle tissue (Figure 4). In the other tumors, even the detailed comparison did not reveal evidence for the presence of copy number alterations that were lacking in the matching myometrial tissue.

## Discussion

There is convincing evidence that recurrent types of *Mediator Subcomplex 12* (*MED12*) mutations and rearrangements of the *HMGA2* locus at 12q14 ~ 15 leading to its transcriptional upregulation represent the two largest groups of independent driver mutations in UL [2,4]. Tumors with *HMGA2*-rearrangements are less frequent than their *MED12*-mutated counterparts [1,2] and preferentially occur in larger, solitary lesions [13]. Moreover, in contrast to UL cells harboring *MED12* mutations, cells with *HMGA2*-rearrangements, as a rule, seem to be able to survive *in vitro* for numerous passages [6].

In a considerable percentage of UL neither chromosomal rearrangements of the *HMGA2* locus nor clonal karyotypic deviations in general nor *MED12* mutations are found. This percentage can be estimated to be approximately 15% and includes cases with rare driver mutations such as e.g. deletions of the *FH*-locus [4]. Nevertheless, even after having deleted these rare mutations there remain some 10% of UL with presumably unknown driver mutations. It seems reasonable to assume that these cases are more frequent among histologically variant UL as e.g. suggested by an epithelioid UL displaying copy number neutral loss of heterozygozity for large parts of its genome [12] that mechanistically has been explained by secondary duplication of the genome of a hyper-haploid tumor. To extend this study and aimed at the detection of copy number alterations confined to the tumor tissue the present study describes the results of a CNV analysis of five UL along with matching myometrial tissue. These possible genomic imbalances might help to uncover rare novel driver mutations. Interestingly, one of the tumors presented with only one small deletion undetectable by classical cytogenetics due to its small size which was absent from the matching myometrium. Nevertheless, this deletion was mapped to the fourth intron of the *HMGA2* gene suggesting it being a driver mutation comparable to the gross and cytogenetically visible rearrangements of *HMGA2* in the cytogenetically abnormal subset of UL.

In contrast, one of the other fibroids revealed multiple genomic deletions of different sizes that should not have escaped detection by cytogenetics. The most plausible explanation for the apparently normal karyotype in that case is that the tumor cells did not proliferate *in vitro* as recently also documented for UL carrying *MED12* mutations [6]. In general, this tumor showed genomic alterations resembling a particular type of genetic damage identified by Stephens et al. [14]. They coined the name chromothripsis to describe massive genomic rearrangements that likely originate from one single catastrophic event. Even earlier, the same phenomenon has been described in breast cancer by Hicks et al. [15] who described the occurrence multiple closely spaced “firestorms” in a large percentage of breast cancer samples where it was found to be associated with an adverse prognosis in both node negative and positive tumors.

However, as a typical sign of “firestorms” or “chromothripsis” the rearrangements are confined to well-circumscribed regions of chromosomes instead of being spread across the whole genome. In contrast, the remaining part of the genome appears to be rather “quiet”. While initially described in hematological malignancies [14,16] and some solid cancers as in particular sarcomas and carcinomas [14,15,17], in a recent study Mehine et al. [4] were able to show that chromothripsis-like rearrangements do also occur in uterine leiomyomas. Again a drastically reduced ability of some leiomyomas to proliferate in vitro may account for the lack of detection of these massive genomic rearrangements by classical cytogenetics though highly complex genomic rearrangements in leiomyomas are occasionally found by conventional cytogenetics. However, the low resolution obtained has certainly hindered to recognize the ‘true complexity’ in these latter cases. As a rule, in chromothripsis the multiple rearrangements are correlated with multiple copy number alterations [18]. Typically, the characteristic changes observed reflect that the process causing this massive but localized breakage has been resolved in the absence of ongoing genomic instability. In the cases presented by Mehine et al. [4] no evidence for atypic or variant UL has been reported. Thus, chromothripsis is not likely to affect exclusively malignant cells and accordingly footprints of resolved chromothripsis do not necessarily accompany malignant transformation. In line with this finding, the case described herein shows chromothripsis apparently in the absence of rearrangements of loci harboring known driver genes and the regions affected by massive rearrangements do not coincide with those shown to be frequently affected by firestorms in breast cancer [15]. While, the tumor presented as a variant leiomyoma upon histological examination it clearly shows that severely disturbed array profiles do not indicate malignancy per se.

As to the molecular pathogenesis of atypical leiomyomas, STUMP, and leiomyosarcomas in a recent paper [19] as well as Zhang et al. [20] were able to show that *MED12* mutations are significantly less common in atypical than in ordinary leiomyomas. Along with our data presented herein and a further case previously reported [12] this may suggest that among the atypical leiomyomas unusual pathways of molecular pathogenesis are overrepresented. In summary, the molecular genetics of atypical leiomyomas constitutes a future challenge for the understanding and diagnosis of these tumors. At least a subset of these tumors may represent variant leiomyomas that had arisen following unorthodox genetic pathways. It remains to be determined if some of these pathways may predispose the variant UL to undergo malignant transformation.

## Conclusions

Apparently, “double negative” leiomyomas, i.e. those lacking clonal cytogenetic derivations as detected by classical cytogenetics as well as *MED12* mutations represent a heterozygous group of tumors. Within that group, CNV arrays offer a suitable tool to identify gross genetic abnormalities which had escaped detection by cytogenetics due to the inability of the cells to survive in vitro. These latter abnormalities may be often occurring among histologically variant leiomyomas thus pointing to rare pathways of molecular pathogenesis. Nevertheless, as such severely disturbed CNV patterns cannot be used to diagnose malignancy.

## Methods

### Tissue samples

Tumor samples and adjacent myometrial tissue snap frozen in liquid nitrogen immediately after surgery (<20 min) were used for array analyses. For all samples corresponding myometrial tissue of the patients has been taken and investigated as well. The study was approved by the local ethics committee (Ethikkommission der Bremer Ärztekammer) and informed written consent was obtained from all patients prior to surgery.

### Histologic examination

For diagnostic purposes tumor samples were fixed in paraformaldehyde (4% in PBS) and processed for paraffin embedding. Tissue sections (1-2 μm thickness) were deparaffinized in xylene, rehydrated through a series of ethanol, and stained with hematoxylin and eosin (H&E) for histologic examination. Classification was based on the World Health Organization classification of tumors of female reproductive organs [21].

### Cytogenetic studies

Chromosome analyses of cell cultures were performed following routine techniques as described earlier [22]. At least 20 metaphases/case were fully karyotyped.

### DNA isolation

DNA from the frozen tissue samples was isolated using the QIAamp DNA Mini Kit (Qiagen, Hilden, Germany) on a QIACube (Qiagen) according to the manufacturer’s instructions.

### PCR and sequencing

For PCR amplification 1000 ng of genomic template DNA were used. Primers to amplify the desired human PCR fragment of the *MED12* gene were those recently described [1,2]. Subsequently, PCR-products were separated by agarose gel-electrophoresis and the desired DNA-fragments/-bands were extracted by a QIAquick Gel Extraction Kit (Qiagen) using a QIACube (Qiagen) according to manufacturer’s instructions. DNA-sequencing of the purified PCR-products was performed by GATC Biotech (Konstanz, Germany).

### RNA isolation

Total RNA from frozen tissue samples was isolated using a RNeasy Mini Kit (Qiagen) on a QIACube (Qiagen) according to manufacturer’s instructions and DNase I digestion was performed.

### cDNA-synthesis

250 ng of total RNA were reverse transcribed with M-MLV reverse transcriptase (Invitrogen, Karlsruhe, Germany), RNase Out (Invitrogen), random hexamers and dNTPs according to the manufacturer’s instructions. RNA was denatured at 65°C for 5 min and subsequently kept on ice for 1 min. After adding the enzyme to the RNA primer mixes, samples were incubated for 10 min at 25°C to allow annealing of the random hexamers. Reverse transcription was performed at 37°C for 50 min followed by inactivation of the reverse transcriptase at 70°C for 15 min.

### Quantitative real-time PCR

Relative quantification of transcription levels was carried out by real-time PCR analyses using the Applied Biosystems 7300 Real-Time PCR system (Applied Biosystems, Darmstadt, Germany). For quantification of HMGA2 mRNA (Hs00171569) a commercially available gene expression assay (Applied Biosystems) was used. HPRT served as endogenous control as described before [23].

### Arrays

CNV (copy number variation) analysis was performed using premade CytoScan HD Arrays (Affymetrix, Santa Clara, CA) consisting more than 2.4 million markers for copy number and approximately 750,000 single nucleotide polymorphisms (SNPs). Enriched gene coverage for cancer and constitutional genes results in a marker-base ratio coverage of 1/384 for ISCA, 1/553 for cancer genes, 1/486 for X-chromosomal genes and 1/659 for 12,000 OMIM genes. The manufacturer’s instructions were followed for labelling of 250 ng DNA, and hybridization. After staining and washing using a GeneChip Fluidics Station 450 (Affymetrix) the arrays were scanned by an Affymetrix 3000 7G scanner. Arrays were analysed through the Affymetrix Chromosome Analysis Suite (ChAS) software (ChAS analysis files for the CytoScan® HD Array version NA32.3). Numbering of map positions was based on hg19 (NCBI Build 37 reference sequence).
